# A nomogram predicting overall survival in patients with non-metastatic pancreatic head adenocarcinoma after surgery: a population-based study

**DOI:** 10.1186/s12885-021-08250-4

**Published:** 2021-05-08

**Authors:** Wenbo Zou, Zizheng Wang, Fei Wang, Gong Zhang, Rong Liu

**Affiliations:** 1 Faculty of Hepato-Pancreato-Biliary Surgery, Chinese PLA General Hospital, No.28 Fuxing Road, Haidian District, Beijing, 100853 China; 2 Institute of Hepatobiliary Surgery of Chinese PLA, Beijing, China; 3 Key Laboratory of Digital Hepetobiliary Surgery PLA, Beijing, China

**Keywords:** Pancreatic head adenocarcinoma, Nomogram, Overall survival, SEER

## Abstract

**Background:**

Pancreatic head adenocarcinoma (PHAC), a malignant tumour, has a very poor prognosis, and the existing prognostic tools lack good predictive power. This study aimed to develop a better nomogram to predict overall survival after resection of non-metastatic PHAC.

**Methods:**

Patients with non-metastatic PHAC were collected from the Surveillance, Epidemiology, and End Results (SEER) database and divided randomly into training and validation cohorts at a ratio of 7:3. Cox regression analysis was used to screen prognostic factors and construct the nomogram. Net reclassification improvement (NRI) and integrated discrimination improvement (IDI) were calculated to evaluate the performance of the model. The predictive accuracy and clinical benefits of the nomogram were validated using the area under the curve (AUC), calibration curves, and decision curve analysis (DCA).

**Results:**

From 2010 to 2016, 6419 patients with non-metastatic PHAC who underwent surgery were collected from the SEER database. A model including T stage, N stage, grade, radiotherapy, and chemotherapy was constructed. The concordance index of the nomogram was 0.676, and the AUCs of the model assessing survival at multiple timepoints within 60 months were significantly higher than those of the American Joint Committee on Cancer (AJCC) 8th staging system in the training cohort. Calibration curves showed that the nomogram had ability to predict the actual survival. The NRI, IDI, and DCA curves also indicated that our nomogram had higher predictive capability and clinical utility than the AJCC staging system.

**Conclusions:**

Our nomogram has an ability to predict overall survival after resection of non-metastatic PHAC and includes prognostic factors that are easy to obtain in clinical practice. It would help assist clinicians to conduct personalized medicine.

**Supplementary Information:**

The online version contains supplementary material available at 10.1186/s12885-021-08250-4.

## Background

Pancreatic cancer has a high mortality rate, and the average 5-year survival rate in the United States is approximately 10% [1]. Because the early symptoms are not obvious, pancreatic cancer is difficult to diagnose and has poor treatment outcomes, which also increase the mortality rate [1, 2]. Currently, radical surgery and adjuvant therapy including radiotherapy and chemotherapy are the main treatment strategies for pancreatic cancer, and radical surgery is the only curative treatment [3, 4]. However, few patients are suitable for surgical treatment because of distant metastases or local invasion at the time of diagnosis [5, 6].

The location of the primary tumour can also affect prognosis and overall survival (OS), and pancreatic head adenocarcinoma (PHAC) is reported to have a significantly shorter OS relative to pancreatic bodytail [7]. Assessment of tumour prognosis primarily relies on the American Joint Committee on Cancer (AJCC) 8th edition staging system [8], which is based on the size and extent of the tumour, the number of metastatic lymph nodes, and the number of distant metastases. The AJCC staging system can guide clinical practice and prognostic analysis to some extent [9]. However, some important prognostic factors are not taken into account in the AJCC staging system, such as age at diagnosis and tumour grade, which reduces its predictive accuracy for some patients. Several prognostic models have been developed to predict the prognosis of pancreatic body/tail tumours [10, 11], although models of non-metastatic pancreatic head tumours still deserve to be explored further. Therefore, a nomogram is needed to predict survival of patients after resection of non-metastatic PHAC and assist with treatment decision-making.

In this study, we screened significant independent factors from the Surveillance, Epidemiology, and End Results (SEER) database to develop a nomogram for modifying the AJCC 8th staging system using Cox regression analysis. The nomogram was constructed for predicting survival in a training cohort and verified in a validation cohort. This nomogram would help assist clinicians to conduct personalized medicine.

## Methods

### Study population and selection criteria

**Fig. 1:**
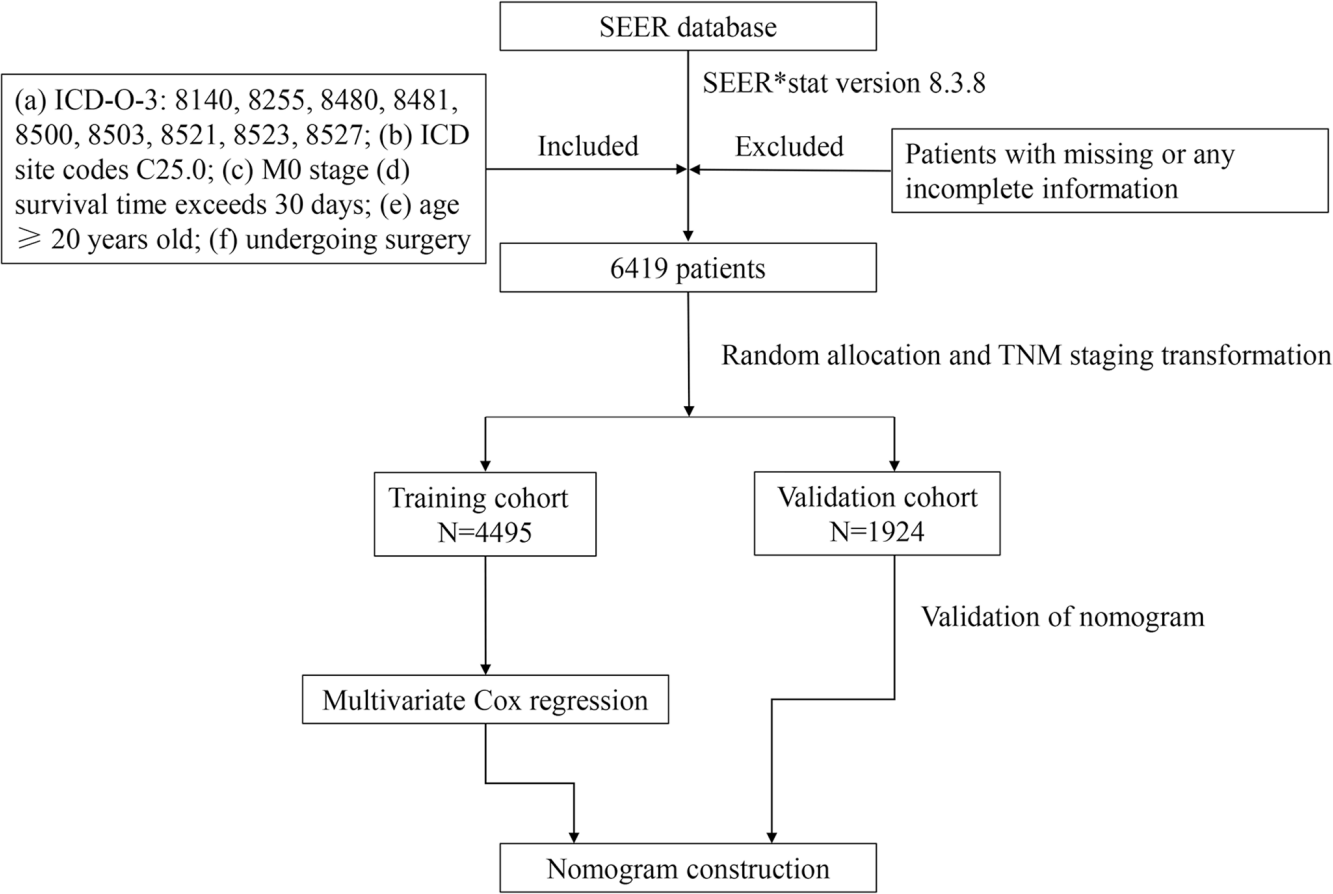
Flowchart showing patient selection and analysis

Patient information was collected from the SEER database of the National Cancer Institute (http://seer.cancer.gov/). After consulting the CS Schema v0204+, we extracted data from 115,227 patients with pancreatic head tumours using SEER*Stat software (version 8.3.8). The inclusion criteria were as follows: (a) International Classification of Diseases for Oncology, Third Edition (ICD-O-3) histology codes 8140, 8255, 8480, 8481, 8500, 8503, 8521, 8523, and 8527; (b) ICD site code C25.0; (c) M0 stage; (d) survival time > 30 days; (e) age ≥ 20 years; (f) underwent surgery; and (g) complete information on race, sex, grade, AJCC staging, radiotherapy, chemotherapy, and the number and status of lymph nodes. Patient lacking any of this information were excluded from the study cohort. Because the original SEER cohort used the 7th edition AJCC staging, we transformed the 7th edition staging to the 8th edition staging for subsequent analysis based on the information about the size and extent of the tumour and the number of positive lymph nodes. The included patients were divided randomly into a training cohort (*n* = 4495) and a validation cohort (*n* = 1924) at a ratio of 7:3 using the ‘caret’ package in R. The training cohort was used to select the prognostic factors and construct the model, and the model performance was verified in the validation cohort. A detailed data-screening process is shown in Fig. 1.

### Statistical analysis

Continuous variables are reported as median (interquartile range) and were analysed by Student’s t test, and categorical variables are reported as counts and proportions and were analysed by chi-squared test for comparisons among groups. Identification of the independent prognostic factors and development of the nomogram model predicting OS was implemented using univariate and multivariate Cox regression analysis. The optimal cut-off values of the lymph node ratio (LNR), tumour size, and total risk score were calculated via X-tile (version 3.6.1). The LNR was defined as the ratio of the number of positive lymph nodes to the number of total examined lymph nodes. We converted continuous variables to categorical variables for further processing and divided patients into ‘low’, ‘medium’, and ‘high’ groups in the training cohort based on the optimal threshold of the total risk score. OS was the primary endpoint and was defined as the time from diagnosis to death or the last follow-up. Kaplan-Meier (KM) survival analysis was performed to depict the capability of individual variables to discriminate OS. With the aim of predicting the OS of patients, a nomogram was finally constructed. The concordance index (C-index) was calculated to evaluate the discrimination of the model, and calibration curves and time-dependent receiver operating characteristic curves within 60 months were created to estimate the predictive capability of the model. The net reclassification improvement (NRI) and integrated discrimination improvement (IDI) were calculated to evaluate the improvement of the model prediction compared to the 8th AJCC staging using the ‘nricens’ and ‘rms’ packages. Decision curve analysis (DCA) is widely applied to evaluate a nomogram with the advantage of integrating patients’ or decision-makers’ preferences into the analysis and is increasingly used in clinical studies. We performed DCA to analyse the clinical benefits of the nomogram compared with those of the 8th AJCC staging system. R (version 4.0.2) and SPSS (version 26.0) were used to complete all statistical analyses in our study, and the main utilized R packages were ‘nricens’, ‘Cschange’, ‘rms’, and ‘timeROC’. A two-sided *p* < 0.05 was considered statistically significant.

## Results

### Demographics and characteristics of the included patients

A total of 6419 patients who underwent resection for non-metastatic PHAC met the criteria and were included in the analysis. Based on the 7:3 ratio of distribution, 4495 and 1924 patients were sorted into the training and validation cohorts, respectively. The median OS in these two cohorts were 16 (8–28) months and 16 (9–27) months, respectively. In the training cohort, most of the patients were 60–80 years old (2984 [66.4%]), the male (2269 [50.5%]) to female (2226 [49.5%]) ratio was 1.02:1, and the predominant race was white (3712 [82.6%]). In terms of pathological diagnosis, most of the patients had T2 stage disease (2798 [62.2%]), N1 stage disease (1823 [40.6%]), and moderately differentiated tumours (2355 [52.4%]). Stage II was the most common clinical stage (1931 [43.0%]). Most patients had tumours > 2.5 cm (2948 [65.6%]). Further, most patients received chemotherapy (3362 [74.8%]) and did not receive radiotherapy (3054 [67.9%]). The median number of retrieved lymph nodes was 17 (12–24), and the median number of positive lymph nodes was 2 (0–4).

To better guide clinical practice, two continuous variables in the training cohort, the LNR and tumour size, were stratified via X-tile (Supplementary Fig. S1 and Table S1). The LNR ranged from 0 to 1, and the tumour size ranged from 0.1 cm to 46 cm. The LNR was grouped into LNR1 (LNR ≤0.20) and LNR2 (LNR > 0.20), and tumour size was classified into group 1 (diameter ≤ 2.5 cm) and group 2 (diameter > 2.5 cm). The baseline clinicopathological characteristics, demographic characteristics, and comparable *p* values are shown in Table 1.

**Table 1:** Demographics and characteristics of patients in Training and Validation cohorts

Characteristics	Training cohort (*n* = 4495)	Validation cohort (*n* = 1924)	*p* value
**Age (year)**	n (%)	n (%)	0.070
< 40	38 (0.8%)	6 (0.3%)	
40–60	1010 (22.5%)	454 (23.6%)	
60–80	2984 (66.4%)	1280 (66.5%)	
≥ 80	463 (10.3%)	184 (9.6%)	
**Gender**			0.102
Female	2226 (49.5%)	910 (47.3%)	
Male	2269 (50.5%)	1014 (52.7%)	
**Race**			0.413
White	3712 (82.6%)	1596 (83.0%)	
Black	411 (9.1%)	186 (9.6%)	
Others	372 (8.3%)	142 (7.4%)	
**8th T stage**			0.100
T1	739 (16.4%)	293 (15.2%)	
T2	2798 (62.2%)	1195 (62.1%)	
T3	786 (17.5%)	376 (19.6%)	
T4	172 (3.8%)	60 (3.1%)	
**8th N stage**			0.931
N0	1321 (29.4%)	561 (29.1%)	
N1	1823 (40.6%)	790 (41.1%)	
N2	1351 (30.0%)	573 (29.8%)	
**Grade**			0.086
Well	476 (10.6%)	222 (11.5%)	
Moderate	2355 (52.4%)	1005 (52.2%)	
Poor	1638 (36.4%)	676 (35.2%)	
Undifferentiated	26 (0.6%)	21 (1.1%)	
**LNR**			0.590
1	3086 (68.7%)	1334 (69.3%)	
2	1409 (31,3%)	590 (30.7%)	
**Tumor size (cm)**			0.909
≤ 2.5	1547 (34.4%)	665 (34.6%)	
> 2.5	2948 (65.6%)	1259 (65.4%)	
**Radiotherapy**			0.647
No/unkown	3054 (67.9%)	1296 (67.4%)	
Yes	1441 (32.1%)	628 (32.6%)	
**Chemotherapy**			0.999
No/unkown	1133 (25.2%)	485 (25.2%)	
Yes	3362 (74.8%)	1439 (74.8%)	
**8th AJCC stage**			0.749
Stage I	1100 (24.5%)	460 (23.9%)	
Stage II	1931 (43.0%)	846 (44.0%)	
Stage III	1464 (32.5%)	618 (32.1%)	
**RLNs**	17 [12–24]	17 [11.25–23]	0.409
**PLNs**	2 [0–4]	2 [0–4]	0.689
**mOS (months)**	16 [8–28]	16 [9–27]	0.539

LNR lymph node ratio, RLNs retrieved lymph nodes, PLNs positive lymph nodes, mOS median overall survival

### Identification and selection of prognostic factors

We performed Cox regression analysis to identify significant prognostic factors correlated with OS. As shown in Table 2, univariate regression analysis revealed that clinicopathological characteristics, including age, grade, T stage, N stage, the LNR, and tumour size, were prognostic risk factors (*p* < 0.001), whereas interventions, including radiotherapy and chemotherapy, were prognostic protective factors (*p* < 0.001). There was no difference in prognosis based on race or gender. The corresponding KM survival curves exhibited the capability of these variables to discriminate the OS in the training cohort (Supplementary Fig. S2).

**Table 2:** Univariate Cox analysis of prognostic factors in the training cohort

Variables	No. of patients	Overall survival	Univariate analysis		
	N	mOS [IQR]	HR	95%CI	*p* value
**Age**			1.24	1.16–1.32	< 0.001
< 40	38	26 [19–35]			
40–60	1010	23 [21–25]			
60–80	2984	21 [20–22]			
≥ 80	463	15 [13–18]			
**Gender**			1.03	0.96–1.11	0.460
Female	2226	21 [20–22]			
Male	2269	21 [20–22]			
**Race**			1.02	0.96–1.08	0.576
White	3712	21 [20–22]			
Black	411	22 [18–25]			
Others	372	20 [18–23]			
**Grade**			1.45	1.37–1.54	< 0.001
Well	476	32 [27–36]			
Moderate	2355	24 [22–25]			
Poor	1638	16 [15–17]			
Undifferentiated	26	18 [11–26]			
**8th T stage**			1.31	1.25–1.38	< 0.001
T1	739	31 [27–34]			
T2	2798	21 [20–22]			
T3	786	16 [15–17]			
T4	172	16 [16–20]			
**8th N stage**			1.44	1.37–1.51	< 0.001
N0	1321	30 [28–33]			
N1	1823	20 [19–22]			
N2	1351	16 [15–17]			
**Tumor size**			1.5	1.39–1.63	< 0.001
Diameter ≤ 2.5	1547	26 [24–29]			
Diameter > 2.5	2948	18 [18–20]			
**LNR**			1.82	1.68–1.96	< 0.001
1	3086	24 [24–25]			
2	1409	15 [14–16]			
**Radiotherapy**			0.71	0.65–0.77	< 0.001
No/unkown	3054	19 [18–20]			
Yes	1441	25 [23–27]			
**Chemotherapy**			0.52	0.48–0.56	< 0.001
No/unkown	1133	12 [11–13]			
Yes	3362	24 [23–25]			

mOS: median overall survival; IQR: interquartile range; HR: Hazard Ratio; CI: Confidence Interval; LNR: lymph node ratio

To improve the application of the model, we further reduced the model variables. T stage is based on tumour size and extension, and the LNR and N stage both describe the metastatic lymph node status. Therefore, we compared the C-indexes of T stage and tumour size and those of the LNR and N stage in the training cohort. The C-index of T stage was 0.556, which was superior to that of tumour size (0.545, *p* = 0.015), and the C-index of N stage was 0.578, which was superior to that of the LNR (0.569, *p* = 0.026). Therefore, we finally developed a better model composed of T stage, N stage, grade, radiotherapy, and chemotherapy. The detailed multivariate Cox regression results are shown in Table 3.

**Table 3:** Multivariate Cox analysis of prognostic factors in the training cohort

	Multivariate analysis		
Variables	HR	95%CI	*p* value
**8th T stage**			
T1	Reference		
T2	1.43	1.28–1.60	< 0.001
T3	1.79	1.57–2.05	< 0.001
T4	2.05	1.67–2.52	< 0.001
**8th N stage**			
N0	Reference		
N1	1.55	1.41–1.70	< 0.001
N2	2.03	1.84–2.25	< 0.001
**Grade**			
Well	Reference		
Moderate	1.39	1.21–1.60	< 0.001
Poor	1.96	1.70–2.26	< 0.001
Undifferentiated	2.75	1.74–4.35	< 0.001
**Radiotherapy (no/unkown vs yes)**	0.81	0.74–0.88	< 0.001
**Chemotherapy (no/unkown vs yes)**	0.49	0.45–0.53	< 0.001

HR: Hazard Ratio; CI: Confidence Interval

### Development and validation of the nomogram predicting overall survival

**Fig. 2:**
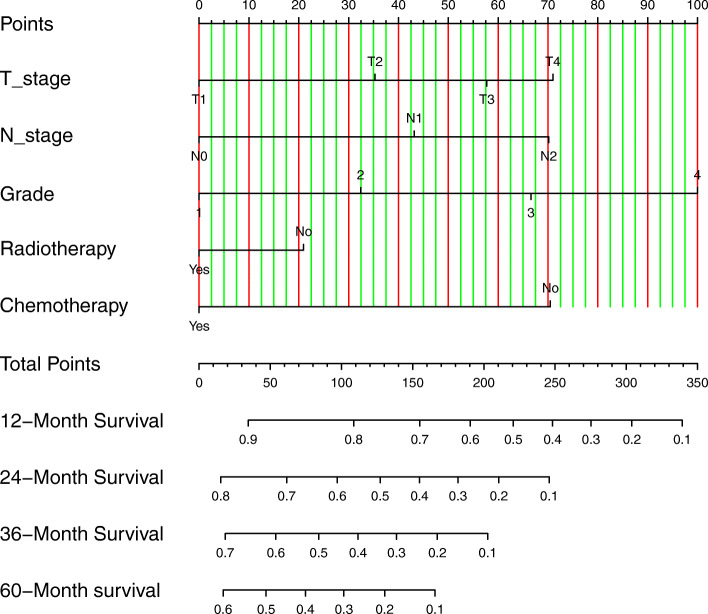
The nomogram predicting overall survival of patients with non-metastatic PHAC. PHAC: pancreatic head adenocarcinoma

Based on this multivariate model, a visualized nomogram was constructed to predict the 1-, 2-, 3-, and 5-year survival rates (Fig. 2). The C-index of the nomogram was 0.676 (95% CI 0.665–0.687), and it had better predictive capability than the AJCC 8th staging system (C-index: 0.581, 95% CI 0.570–0.591, *p* < 0.001) in the training cohort. Similarly, in the validation cohort, the C-index was 0.667 (95% CI 0.649–0.684), and it also had better predictive capability than the AJCC 8th staging system in this cohort (C-index: 0.566, 95% CI 0.548–0.584, *p* < 0.001). The NRI and IDI were also calculated to demonstrate the predictive accuracy of the nomogram. In the training cohort, the NRIs based on bootstrapping for OS at 1, 2, 3, and 5 years were 0.305 (95% CI 0.248–0.357), 0.227 (95% CI 0.172–0.284), 0.057 (95% CI 0.003–0.108), and 0.132 (95% CI 0.085–0.197), respectively. The IDIs for OS at 1, 2, 3, and 5 years were 0.050, 0.063, 0.056, and 0.045, respectively (*p* < 0.001) (Table 4). The results were also verified in the validation cohort (Table 4). Therefore, the nomogram had better predictive accuracy for OS at 1, 2, 3, and 5 years than the AJCC 8th staging system.

**Table 4:** The NRIs, IDIs, and C-indexes of the nomogram and AJCC staging system in OS prediction

	Training cohort			Validation cohort		
Index	Estimate	95%CI	*p* value	Estimate	95%CI	*p* value
NRI (vs. the AJCC staging)						
For 12-month OS	0.305	0.248–0.357		0.212	0.155–0.332	
For 24-month OS	0.227	0.172–0.284		0.163	0.104–0.258	
For 36-month OS	0.057	0.003–0.108		0.087	0.024–0.232	
For 60-month OS	0.132	0.085–0.197		0.186	0.089–0.263	
IDI (vs. the AJCC staging)						
For 12-month OS	0.05		***p*** **< 0.001**	0.044		***p*** **< 0.001**
For 24-month OS	0.063		***p*** **< 0.001**	0.059		***p*** **< 0.001**
For 36-month OS	0.056		***p*** **< 0.001**	0.055		***p*** **< 0.001**
For 60-month OS	0.045		***p*** **< 0.001**	0.046		***p*** **< 0.001**
C-index						
The nomogram	0.676	0.665–0.687		0.667	0.649–0.684	
The AJCC staging	0.581	0.570–0.591		0.566	0.548–0.584	
Change	0.095		***p*** **< 0.001**	0.101		***p*** **< 0.001**

AJCC*:* American Joint Committee on cancer; OS: overall survival; IDI: integrated discrimination improvement; NRI: net reclassification improvement; C-index: concordance index; CI: Confidence Interval

**Fig. 3:**
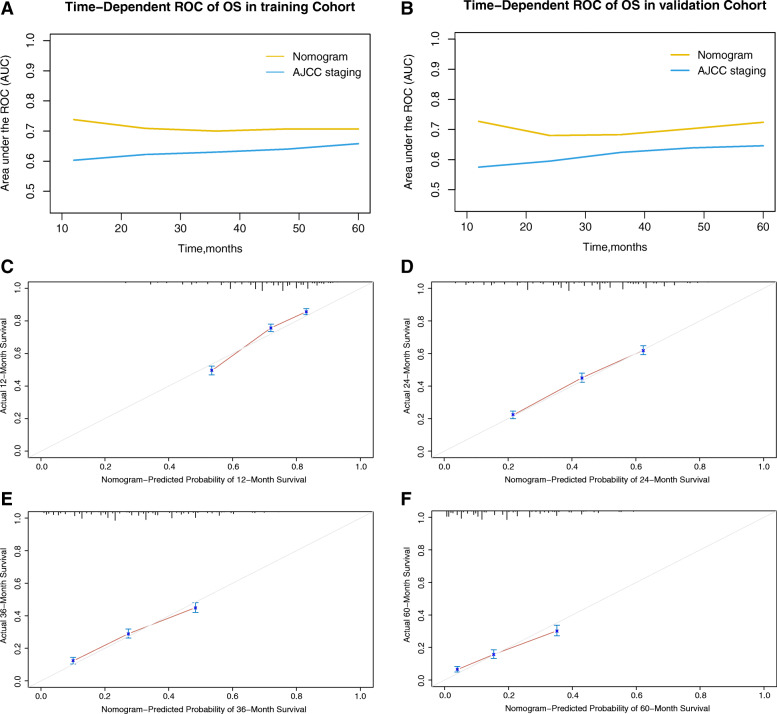
**a-b** Comparison of the time-dependent AUCs between the nomogram and the AJCC staging system in the training and validation cohorts. **c-f** Calibration curves showing the probability of 12-, 24-, 36-, and 60-month overall survival of the model prediction and the observed survival in the training cohort. AUC: area under the curve; AJCC: American Joint Committee on Cancer

The time-dependent AUCs of the nomogram for predicting OS within 60 months in the training and validation cohorts are shown in Fig. 3A-B. The AUCs were significantly higher in both cohorts than those of the AJCC 8th staging system, indicating that the model had favourable discrimination. The calibration curves for survival probability showed that the nomogram prediction had good consistency with the actual observation in the training cohort at 12, 24, 36, and 60 months (Fig. 3C-F); the validation cohort showed similar results (Supplementary Fig. S3).

**Fig. 4:**
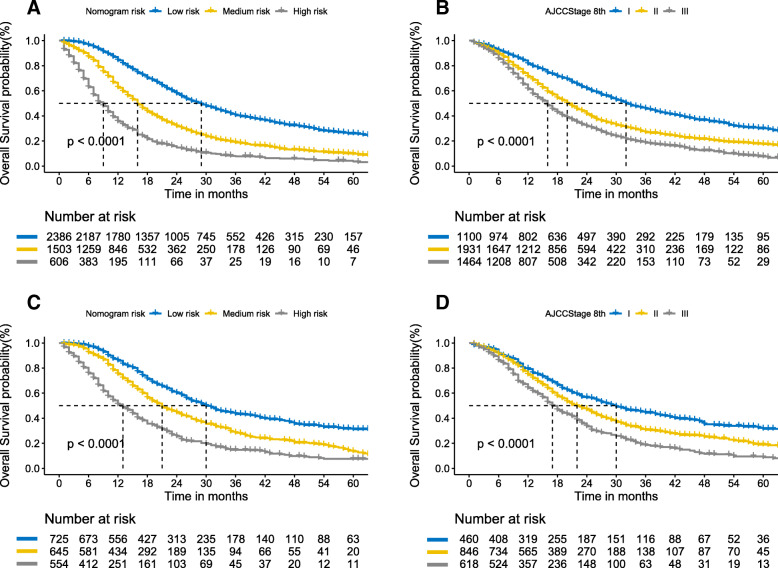
**a-d** Kaplan-Meier curves of overall survival for patients based on the nomogram risk grouping and the AJCC staging system in the two cohorts. AJCC: American Joint Committee on Cancer

Based on the optimal cut-off value of the total nomogram score obtained using X-tile, we divided all patients into three risk groups: ‘low risk’ (≤138), ‘medium risk’ (139–195), and ‘high risk’ (> 195). The detailed risk score is shown in Supplementary Table S2. The KM survival curves showed that the nomogram risk grouping had better discrimination for OS than the AJCC 8th staging system (Fig. 4, Supplementary Table S3).

### Clinical utility analysis of the nomogram

**Fig. 5:**
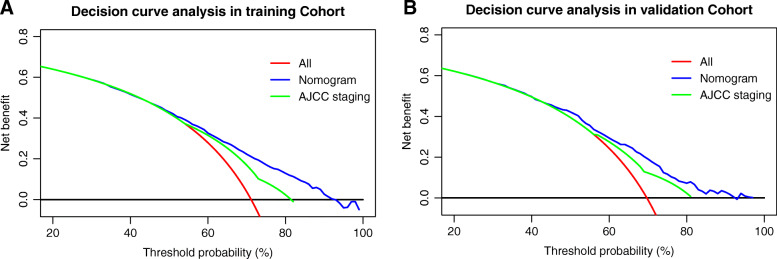
**a-b** Decision curve analysis showed clinical benefits of the model predicting overall survival in the training and validation cohorts

To explore the clinical utility of the nomogram, we performed DCA. The results showed that the nomogram had better net benefits than the AJCC 8th staging system in both the training and validation cohorts (Fig. 5A-B), suggesting that the clinical utility of the nomogram is better than that of the AJCC 8th staging system.

## Discussion

Few patients with PHAC are able to undergo surgical resection, and even patients who do undergo resection have a low 5-year survival rate. Therefore, there is an urgent need for a nomogram to predict survival for these patients. Since patients with metastatic PHAC are less likely to undergo surgery, we are more interested in patients who undergo resection of non-metastatic PHAC and desired to develop a predictive nomogram for this cohort.

Here, we performed a population-based study involving data from 6419 patients who underwent resection of non-metastatic PHAC from the SEER database. After assessing multiple variables, including age, gender, and AJCC staging, we finally developed a nomogram including five independent prognostic factors (T stage, N stage, grade, radiotherapy, and chemotherapy) that predicted OS at 1, 2, 3, and 5 years after resection with high accuracy. Importantly, these prognostic factors are easy to obtain in clinical practice. The results of our analyses, including the AUC, NRI, and IDI, showed that our model had better accuracy for predicting survival than the AJCC 8th staging system, and DCA showed the model had higher clinical benefits than the AJCC staging system. The calibration curves also showed good discrimination and calibration capabilities.

Previous studies have proposed various significant prognostic factors for patients with pancreatic cancer, such as age, lymph node metastasis, and biochemical indicators [10, 12–14]. Similarly, our study also demonstrated that age, T stage, N stage, grade, the LNR, and tumour size were significant risk factors; radiotherapy and chemotherapy were strong protective factors. He et al. revealed that high-grade classification indicates poor prognosis in adenocarcinoma of the pancreatic body and tail [11]. In our study, the high-grade classification was a strong prognostic risk factor of PHAC, so we included tumour grade into this model. The LNR was recently implemented as a more accurate assessment of lymph node metastasis to modify N stage and has been used to predict prognosis and guide clinical practice for patients with pancreatic cancer [15–18]. In our study, the LNR was an independent prognostic factor in Cox regression analysis. The cut-off value of 0.2 was also appropriate and further applied to the controversial problem of threshold. We then further compared the predictive abilities of the LNR and N stage by comparing their C-indexes. Interestingly, we found no significant difference. However, N stage is a more accessible factor than the LNR. Therefore, the LNR may not be superior to N stage for predicting survival in patients with non-metastatic PHAC after surgery.

Radical surgery and chemoradiotherapy are currently the main treatment strategies for patients with pancreatic cancer [1]. A phase III clinical trial revealed that adjuvant chemoradiotherapy can significantly increase the R0 resection rate and prolong survival [19]. Recent studies have also reported that chemoradiotherapy is a key protective factor in advanced pancreatic cancer [20–22]. Administration of adjuvant chemoradiotherapy can significantly improve the survival of PHAC patients. In our study, we demonstrated that radiotherapy and chemotherapy were strong protective factors in this large study population. Therefore, our nomogram included these interventions. The nomogram had better predictive capability than the AJCC 8th staging system. Therefore, physicians can use postoperative indicators and these treatment measures to assess patient prognosis and guide further treatment.

Although our model had good performance for predicting survival, some limitations must also be noted. First, the data were collected from the SEER database, and this was a retrospective study with inherent defects. Further, some significant prognostic factors, such as the resection margin, the level of carbohydrate antigen 19–9, and some physical indices, were not available in the SEER database. Second, the cut-off value of partial variables in our study were calculated by X-tile, and the wide application of the threshold requires further confirmation. Further large prospective studies to confirm the effectiveness of our prognostic model are also needed.

## Conclusions

We developed and validated a nomogram that has high accuracy in predicting OS in patients after resection of non-metastatic PHAC. All of the included prognostic factors are easy to obtain. This nomogram could promote the development of a novel staging system and assist clinicians in conducting personalized clinical treatment.

## Supplementary Information

**Additional file 1: Fig. S1.** Cut-off value of the LNR and tumor size were calculated from the training cohort. A, B LNR. C, D tumor size. In the plot A and C, red represents the negative correlation between the variables and survival, whereas green represents a positive correlation. In the plot B and C, it shows the cut-off value of variables and numbers of patients in subgroups. **Fig. S2.** Kaplan-Meier curves of patients based on obtained prognostic variables. A age. B race. C sex. D T stage. E N stage. F grade classification. G LNR. H tumor size. I radiotherapy. J chemotherapy. **Fig. S3.** Calibration curves showed the probability of 12-, 24-, and 36-, and 60- year OS between the model prediction and the practical observation in the validation cohort. **Table S1.** The optimal cut-off value of LNR and tumor size in training cohort. **Table S2.** Nomogram score of prognostic variables. **Table S3.** Overall Survival probability and Median Survival Time of Prognostic Nomogram Score and current criteria in the two cohorts.

## Data Availability

The data that support the findings of this study are available from the corresponding author upon reasonable request, and were publicly available and obtained from the Surveillance, Epidemiology, and End Results database (http://seer.cancer.gov/).

## Abbreviations

AJCC: American Joint Committee on Cancer
AUC: Area under the curve
C-index: Concordance index
CI: Confidence Interval
DCA: Decision curve analysis
HR: Hazard Ratio
OS: Overall survival
ICD: International Classification of Diseases
IDI: Integrated discrimination improvement
KM: Kaplan-Meier
LNR: Lymph node ratio
NRI: Net reclassification improvement
PHAC: Pancreatic head adenocarcinoma
SEER: Surveillance, Epidemiology, and End Results database
