# Supplementary material for: A nomogram predicting overall survival in patients with non-metastatic pancreatic head adenocarcinoma after surgery: a population-based study
Source: BMC Cancer. 2021 May 8;21:524. doi: 10.1186/s12885-021-08250-4 (PMC8106852; doi:10.1186/s12885-021-08250-4)
Supplement: Supplementary file 1 — Additional file 1: Fig. S1. Cut-off value of the LNR and tumor size were calculated from the training cohort. A, B LNR. C, D tumor size. In the plot A and C, red represents the negative correlation between the variables and survival, whereas green represents a positive correlation. In the plot B and C, it shows the cut-off value of variables and numbers of patients in subgroups. Fig. S2. Kaplan-Meier curves of patients based on obtained prognostic variables. A age. B race. C sex. D T stage. E N stage. F grade classification. G LNR. H tumor size. I radiotherapy. J chemotherapy. Fig. S3. Calibration curves showed the probability of 12-, 24-, and 36-, and 60- year OS between the model prediction and the practical observation in the validation cohort. Table S1. The optimal cut-off value of LNR and tumor size in training cohort. Table S2. Nomogram score of prognostic variables. Table S3. Overall Survival probability and Median Survival Time of Prognostic Nomogram Score and current criteria in the two cohorts. [file 12885_2021_8250_MOESM1_ESM.docx]

Supplementary Materials

**Title:**

A nomogram predicting overall survival in patients with non-metastatic pancreatic head adenocarcinoma after surgery: a population-based study

**Supplementary Figure S1**


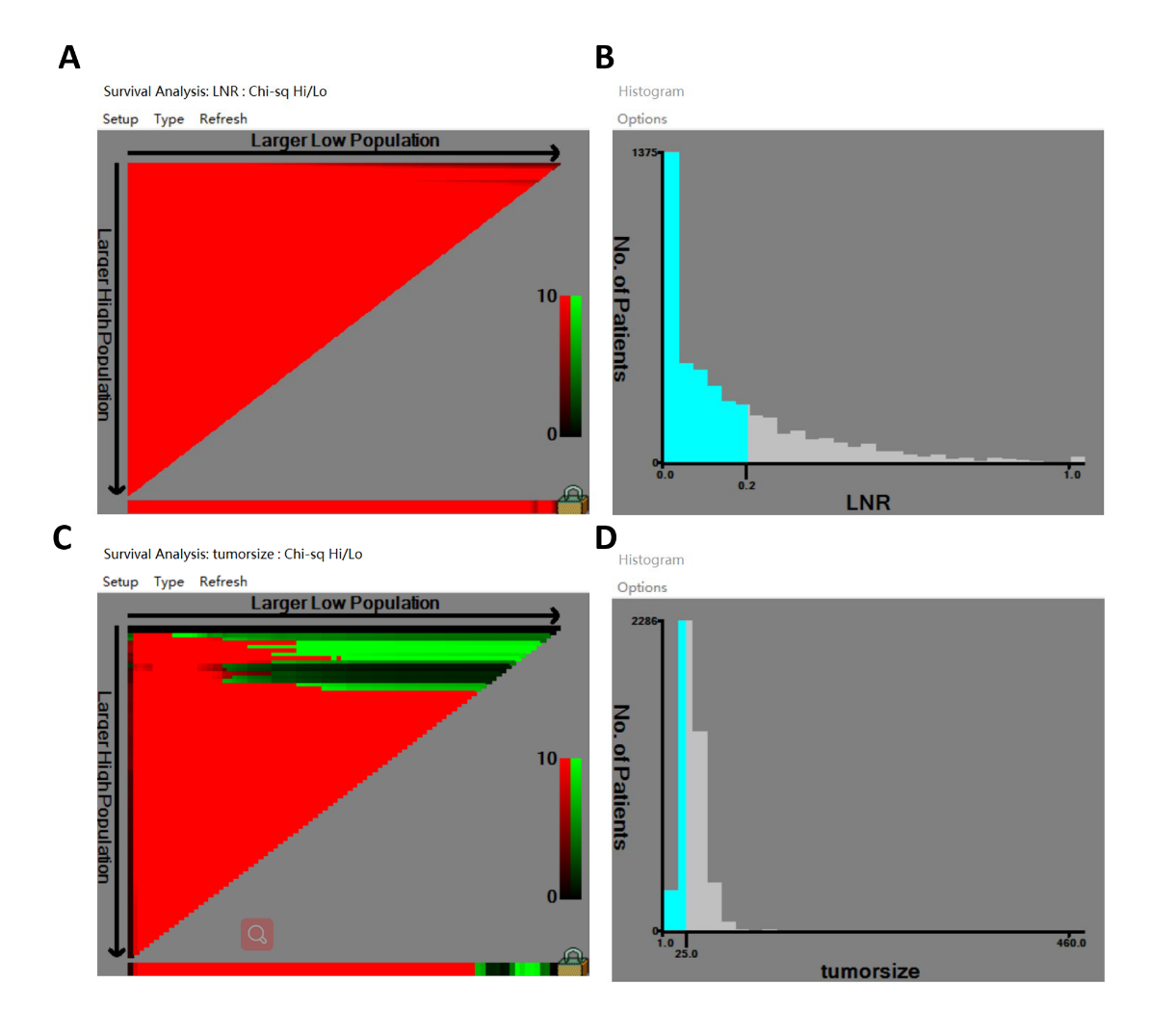


Supplementary Figure S1. Cut-off value of the LNR and tumor size were calculated from the training cohort. A, B LNR. C, D tumor size. In the plot A and C, red represents the negative correlation between the variables and survival, whereas green represents a positive correlation. In the plot B and C, it shows the cut-off value of variables and numbers of patients in subgroups.

**Supplementary Figure S2**

**
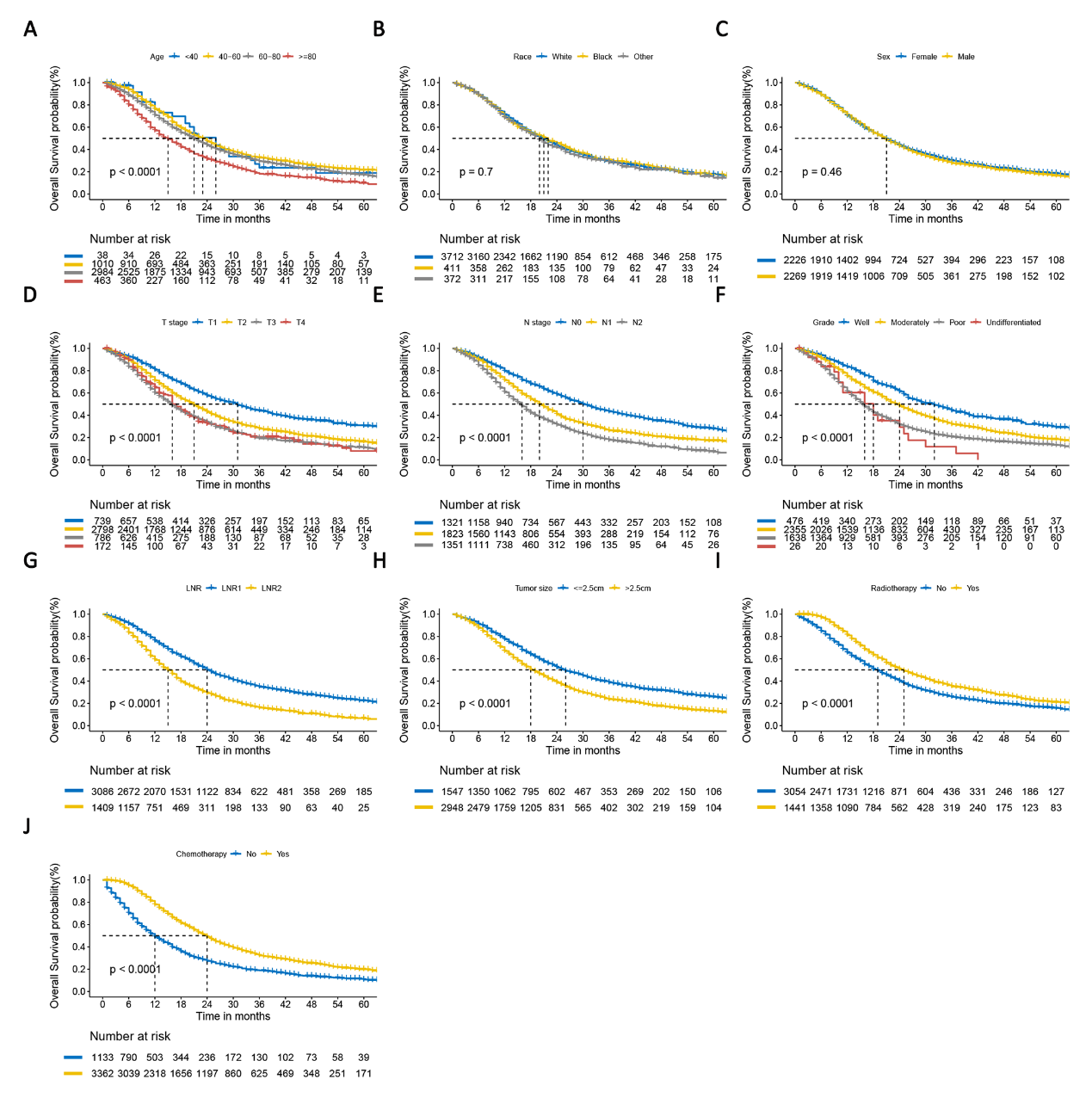
**

Supplementary Figure S2. Kaplan-Meier curves of patients based on obtained prognostic variables. A age. B race. C sex. D T stage. E N stage. F grade classification. G LNR. H tumor size. I radiotherapy. J chemotherapy.

**Supplementary Figure S3**

**
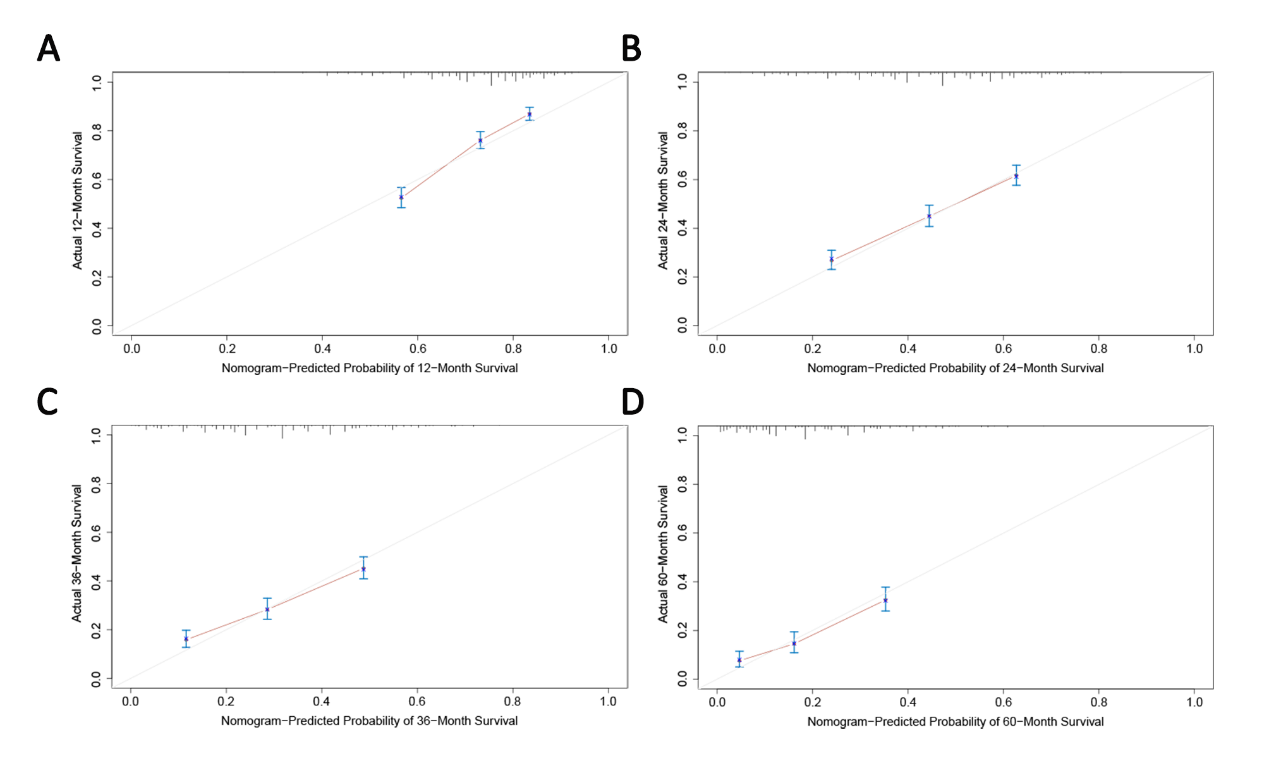
**

Supplementary Figure S3. Calibration curves showed the probability of 12-, 24-, and 36-, and 60- year OS between the model prediction and the practical observation in the validation cohort.

**Supplementary Table S1**

| **Table S1** The optimal cut-off value of LNR and tumor size in training cohort. | | | | |
| --- | --- | --- | --- | --- |
| Variables | Numbers of patients | DR | RR | *p* value |
| LNR |  |  |  | <0.001 |
| LNR≤0.20 | 3086 | 57.74% | 1.00 |  |
| LNR>0.20 | 1409 | 74.24% | 1.29 |  |
| Tumor size(cm) |  |  |  | <0.001 |
| Diameter≤2.5 | 1547 | 55.79% | 1.00 |  |
| Diameter > 2.5 | 4495 | 66.66% | 1.19 |  |
| *DR* dead rate, *RR* relative risk | | | | |

**Supplementary Table S2**

| **Table S2** Nomogram score of prognostic variables | |
| --- | --- |
| **Variables** | **Points** |
| **8th T stage** |  |
| T1 | 0 |
| T2 | 24 |
| T3 | 49 |
| T4 | 73 |
| **8th N stage** |  |
| N0 | 0 |
| N1 | 34 |
| N2 | 69 |
| **Grade** |  |
| well | 0 |
| moderate | 33 |
| poor | 67 |
| Undifferentiated | 100 |
| **Radiotherapy** |  |
| no/unkown | 21 |
| yes | 0 |
| **Chemotherapy** |  |
| no/unkown | 68 |
| yes | 0 |

**Supplementary Table S3**

| **TableS3** Overall Survival probability and Median Survival Time of Prognostic Nomogram Score and current criteria in the two cohorts | | | | |
| --- | --- | --- | --- | --- |
| **Current Criteria** | **Stage** | **Year-rate and Median  Survival Time (month)** | **Training Cohort** | **Validation Cohort** |
| Prognostic Nomogram Score | <=138 | 12-month | 84.2% | 85.9% |
|  |  | 24-month | 57.6% | 60.0% |
|  |  | 36-month | 40.4% | 43.4% |
|  |  | 48-month | 32.5% | 35.5% |
|  |  | 60-month | 25.9% | 31.2% |
|  |  | Median Survival Time | 29[27-31] | 30[27-34] |
|  | 138-195 | 12-month | 62.5% | 75.1% |
|  |  | 24-month | 31.6% | 44.4% |
|  |  | 36-month | 18.8% | 28.4% |
|  |  | 48-month | 13.1% | 20.5% |
|  |  | 60-month | 9.8% | 13.5% |
|  |  | Median Survival Time | 16[16-17] | 21[19-24] |
|  | >195 | 12-month | 35.4% | 51.0% |
|  |  | 24-month | 14.7% | 25.4% |
|  |  | 36-month | 7.8% | 14.8% |
|  |  | 48-month | 6.0% | 9.9% |
|  |  | 60-month | 3.7% | 76.2% |
|  |  | Median Survival Time | 9[8-10] | 13[12-15] |
| AJCC stage | I | 12-month | 81.4% | 79.3% |
|  |  | 24-month | 61.5% | 56.5% |
|  |  | 36-month | 45.7% | 44.5% |
|  |  | 48-month | 36.8% | 35.2% |
|  |  | 60-month | 30.1 | 31.5% |
|  |  | Median Survival Time | 32[30-36] | 22[26-38] |
|  | II | 12-month | 71.2% | 74.6% |
|  |  | 24-month | 41.4% | 46.9% |
|  |  | 36-month | 26.8% | 30.7% |
|  |  | 48-month | 21.6% | 25.2% |
|  |  | 60-month | 17.5% | 19.0% |
|  |  | Median Survival Time | 20[20-22] | 22[20-25] |
|  | III | 12-month | 61.3% | 64.1% |
|  |  | 24-month | 31.7% | 33.5% |
|  |  | 36-month | 18.6% | 18.6% |
|  |  | 48-month | 12.5% | 11.5% |
|  |  | 60-month | 7.6% | 8.9% |
|  |  | Median Survival Time | 16[15-17] | 17[16-19] |
| *AJCC* American Joint Committe on cancer | | |  |  |
